# Paternal effects in the initiation of migratory behaviour in birds

**DOI:** 10.1038/s41598-021-81274-9

**Published:** 2021-02-02

**Authors:** V. Méndez, J. A. Gill, B. Þórisson, S. R. Vignisson, T. G. Gunnarsson, J. A. Alves

**Affiliations:** 1 South Iceland Research Centre, University of Iceland, Laugarvatn, 840 Iceland; 2 School of Biological Sciences, University of East Anglia, Norwich, NR4 7TJ UK; 3 Sudurnes Science and Learning Center, Sandgerði, 245 Iceland; 4 Department of Biology and CESAM – Centre for Environmental and Marine Studies, University of Aveiro, 3910-193 Aveiro, Portugal

**Keywords:** Animal migration, Behavioural ecology

## Abstract

What determines why some birds migrate and others do not? This question is fundamental to understanding how migratory systems are responding to environmental changes, but the causes of individual migratory behaviours have proven difficult to isolate. We show that, in a partially migratory population of Eurasian oystercatchers (*Haematopus ostralegus*), the migratory behaviour of progeny follows paternal but not maternal behaviour, and is unrelated to timing of hatching or fledging. These findings highlight the key role of social interactions in shaping the migratory behaviour of new generations, and thus the spatio-temporal distribution of migratory populations.

## Introduction

Animal migration has long been one of the most fascinating of natural phenomena. Migratory behaviour typically arises in seasonal environments, allowing individuals to exploit seasonal peaks of resource abundance in distinct locations across the world^1^. However, rapid shifts in the distribution and migration phenology of many migratory species^2,3^, and the consequent challenges to site-based conservation strategies^4^, have highlighted the urgent need to determine the processes influencing individual migratory behaviour in order to understand and predict species’ responses to environmental change.

Most migratory avian species have a geographically broad non-breeding range, and individuals from the same breeding locations frequently travel to different parts of the non-breeding range^5^. Migratory individuals are typically highly consistent and repeatable in the locations they occupy within- and between-years^3,6–8^, and individual fitness can be influenced by the conditions experienced on the locations occupied at each point in the annual cycle^9^. Consequently, the processes influencing the initiation of individual migratory behaviour (e.g. distance and direction travelled) are likely to be major drivers of subsequent individual fitness and population distribution^3^. Conditions experienced during the early stages of life, such as hatching date and the associated time available for chick growth, could potentially influence the initiation of individual migratory behaviour. For example, late-hatched chicks and/or those with slow growth rates could face constraints in preparing for migration, such as late-season resource constraints and shorter pre-migratory periods in which to acquire the necessary fat reserves^10^, or have fewer opportunities to join experienced conspecifics during the post-fledging period^3,11,12^. Hence, these juveniles could be more likely to migrate later in the autumn or to remain closer to the breeding grounds during winter than those hatched earlier in the season. The contributions of social information to the initiation of migratory behaviour may be particularly important in species with strong flocking behaviour^13^. However, juvenile migratory behaviour could also be influenced by parental migratory behaviour, particularly in systems where there is an extended period of post-fledging parental care of juveniles^14,15^ or where migration takes place in family parties^16^.

The development of individual migratory behaviour is a complex issue to unravel because it requires either experimental manipulations^17^ or individuals from the same families to be tracked across migratory ranges, in systems with substantial variation in breeding phenology and migratory behaviour. In the population of Eurasian oystercatchers breeding in Iceland, individuals show marked differences in migratory behaviour, with ~ 30% of the population wintering in Iceland (hereafter referred as residents, but note that short-distance movements within Iceland can occur) and the remainder migrating at least 750 km over open ocean to coastal sites throughout western Europe (hereafter referred as migrants)^18,19^. As adult site-fidelity to breeding and non-breeding location is high^19,20^, individual migratory behaviour is likely to be determined in early life. In this population, timing of breeding of migrants is influenced greatly by annual variation in weather conditions, while residents are more consistent in their timing of breeding^21^. Oystercatchers are long-lived and monogamous, retaining the same mate and nesting site from year to year^22,23^. Unlike most other shorebirds (though typical of most birds), young oystercatchers are fed by their parents throughout the growing period, and thus growth rates could be influenced by parental provisioning effort. A particularly relevant feature of this population is the relatively high proportion of pairs of mixed migratory behaviour. In ~ 20% of pairs both members are resident, in ~ 46% of pairs both members are migrant and ~ 34% of pairs have one migrant and one resident member^19^. This system therefore provides an ideal opportunity to identify the influence of timing, growth and parental migratory behaviour in the migratory behaviour adopted by juveniles.

## Results

**Figure 1:**
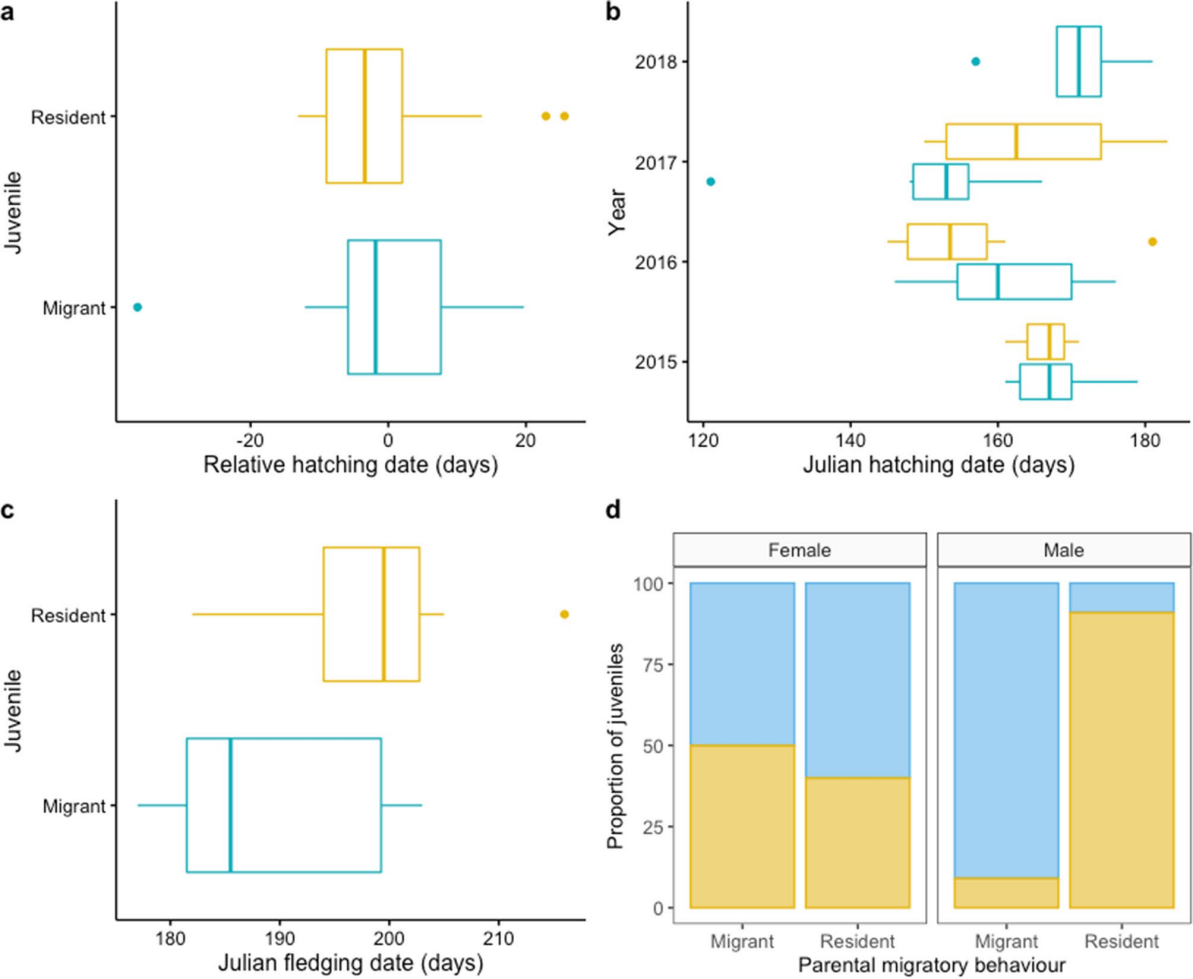
Potential factors determining the initiation of individual migratory behaviour in a partially migrant population. **a** Relative egg-hatching date of migrant (n = 31) and resident (n = 19) juveniles. **b** Annual variation in Julian egg-hatching date of migrant (n = 31) and resident (n = 19) juveniles. **c** Julian fledging dates of migrant (n = 6) and resident (n = 8) juveniles. **d** Proportion of migrant and resident juveniles grouped by their maternal (n = 21) and paternal (n = 22) migratory behaviour. Colour indicates juvenile migratory behaviour (blue = migrant, yellow = resident). The line inside the boxplot represents the median and the whiskers represent scores outside the inter-quartile range (middle 50%).

Whether individual chicks subsequently became residents or migrants was not associated with their hatch dates, measured as either relative (i.e. mean centred hatching date for each year; Table 1, Fig. 1a) or absolute hatch dates (Table 1), even though absolute hatch dates varied quite substantially among years (Fig. 1b). Similarly, pre-fledging growth parameters (asymptotic value of foot length and body mass (*y*_∞_), growth rate constant (*k*) and age at the inflection point (*T*_i_)) did not differ between individuals that became resident or migrant (Supplementary Table S3), and fledging dates (despite the smaller sample size) did not appear to influence the likelihood of becoming migrant or resident (Table 1; Fig. 1c). However, whether individual chicks subsequently became residents or migrants was strongly associated with paternal but not maternal migratory behaviour (Table 2; Fig. 1d), with fathers and their offspring sharing migratory behaviour in ~ 90% of cases while mothers’ behaviour had no association with offspring behaviour (Table 2). Seven chicks fledged from pairs with one resident and one migrant parent, and in all seven cases the chicks adopted the same migratory behaviour as their fathers (Supplementary Table S1)**.** This paternal effect did not appear to result from differences in breeding phenology between migrant and resident fathers (mean relative hatch date of chicks from migrant fathers = − 4.22 ± 3.94 SE and from resident fathers = − 0.69 ± 3.60 SE, *t* = − 0.66, *df* = 19.84, *p* = 0.52; mean absolute hatch date of chicks from migrant fathers = 155.82 ± 4.70 SE and from resident fathers = 159.18 ± 3.90 SE, *t* = − 0.55

**Table 1:** Results from the generalised linear models testing for phenological effects on the likelihood of juveniles becoming migrant or resident. Note that the standard error associated with the estimate for 2018 is large as there are no resident juveniles from that year.

	Predictor	Estimate	Std. error	z value	*p*
1)	(Intercept)	− 0.49	0.29	− 1.69	0.09
	Relative hatching date	− 0.01	0.03	− 0.18	0.85
2)	(Intercept)	− 2.06	5.27	− 0.39	0.70
	Absolute hatching date	0.01	0.03	0.23	0.82
	Year^a^				
	2016	1.33	0.89	1.50	0.13
	2017	− 0.07	0.98	− 0.08	0.94
	2018	− 16.74	1768.36	− 0.01	0.99
3)	(Intercept)	− 14.30	10.60	− 1.35	0.18
	Fledging date	0.08	0.05	1.37	0.17

^a^Reference year: 2015.

, *df* = 19.34, *p* = 0.59).

**Table 2:** Results from the generalised linear models testing parental migratory effect on juvenile migratory behaviour. The estimates represent the log odds of adults having resident juveniles (non-reference category in the response variable).

Parent	Parental migratory behaviour	Estimate	Std. Error	z value	Pr( >|z|)
Father	Migrant	− 2.30	1.05	− 2.19	0.028
	Resident	4.60	1.48	3.10	0.002
Mother	Migrant	0	0.50	0	1.000
	Resident	− 0.41	1.04	− 0.39	0.697

## Discussion

Tracking of individual Icelandic oystercatcher chicks to their non-breeding locations has revealed that most chicks adopt the migratory behaviour of their fathers, but not their mothers, and that time- or resource-constraints during the pre-fledging period appear to have little influence on subsequent migratory behaviour of these chicks. Given the lack of evidence of innate (genetic) control of migratory destinations^17,24^, and the contribution of both partners to parental care during pre-fledging period in *Haematopus* sp.^25,26^, what mechanism could produce such strong paternal but not maternal effects?

Our results suggest that the migratory behaviour of individual oystercatchers is linked to social interactions during the post-fledging period, specifically the paternal bond. In monogamous, single-clutch shorebird species**,** like oystercatchers, mothers commonly depart before, or at, chick fledging, while fathers often provided parental care for longer, even in species without direct parental provisioning^27,28^. This extended maintenance of the paternal bond may be the underlying driver of the link between paternal and juvenile migratory behaviour. Despite being able to fly and feed independently, begging behaviour in fully-fledged juvenile oystercatchers has been observed to extend for several months after fledging^29^, suggesting that parents (most likely fathers^27^) may provide an extended period of parental care. For residents, paternal care that extends beyond the period when migratory individuals leave the breeding areas would mean that their offspring would likely also become residents. For migrants, paternal care may extend only into the period when pre-migratory flocks form, as shorebirds (including oystercatchers) typically do not migrate in family parties or remain together during winter^7,30–33^. However, juveniles in pre-migratory flocks are likely to mix with and learn from experienced individuals that share the paternal migratory behaviour. Thus, the social cues experienced by offspring during the post-breeding period may be a key mechanism in the initiation and development of individual migratory behaviour. While we were unable to detect any significant effect of fledging date on migratory behaviour, virtually all of the earliest fledgers in our population went on to become migrants, and we cannot rule out the possibility that very late-fledging individual lack the time or resources to undertake a migratory journey irrespective of paternal behaviour.

Migratory individuals (including oystercatchers) are typically site-faithful^3,24^ and the quality of sites they occupy can influence individual fitness and population-level processes^31,34^. Consequently, the processes determining juvenile migratory and settlement decisions are likely to be key drivers of the evolution and maintenance of migratory routes and ranges (including partial migration), and of migratory range change and the associated implications for protected area networks^4^. Our findings suggest that the social interactions experienced by individuals can directly influence the ontogeny of their migratory behaviour, and that the extent and timing of parental care can be key in shaping individual access to these social interactions.

## Methods

### Tracking of individuals

From 2015 to 2018, incubating adults were caught on the nest using a spring-trap, measured and individually marked with coloured leg-rings, and feather samples were collected for stable isotope analysis^19^. Chicks were first caught, metal ringed and measured in the nest and then fitted with individual combinations of colour-rings once tarsi had grown to a sufficient length (around 2 weeks old). Through a network of volunteer observers reporting sightings of marked individuals across the wintering range, the migratory behaviour of 227 of the 615 colour-marked adults and 50 of the 377 colour-marked chicks have been identified (Supplementary Table S2). The winter period (during which only resident individuals are likely to be present in Iceland) was defined from the beginning of October to the end of February. No migrant individuals have been recorded in Iceland after September and the earliest returning migrants have been observed during the first week of March (personal observations). In addition, for 353 marked adults, migratory behaviour has been determined using a discriminant function analysis of stable isotope ratios (*δ*^13^C and *δ*^15^N), after calibration using the isotopic signatures of those individuals that were observed during winter within or outside Iceland^19^ (Supplementary Table S2). For adults of juveniles with known migratory behaviour (n = 40 adults), their migratory behaviour was determined by either winter resighting (18; males = 11; females = 7) or stable isotope ratios (22; males = 10; female = 12). In three cases, the migratory behaviour of two juveniles originating from the same brood was determined (both adopted the same migratory behaviour). The discriminant function analysis used for the classification of migratory behaviour given the stable isotope ratio has an error rate of only 9%, and only individuals with ≥ 67% of assigned probability to one of the migratory behaviours were considered in this study^19^. Since this analysis, 73 additional individuals with assignment probability of ≥ 0.67 have thus far been recorded in winter and all showed the assigned migratory behaviour.

### Hatching date, growth and fledging date

We surveyed study areas in South, West and North-West Iceland from the beginning (mid-April) of the breeding season to the end (July) to search for nests^19^. For nests found during incubation, we used egg flotation methods^35^ to predict hatching date, assuming 28 days of incubation starting when last egg was laid, thus ensuring that newly hatched chicks would still be in the nest cup for initial measurement and ringing, and noted this as Julian hatching date.

Oystercatcher parents remain in the vicinity of the nest after chick hatching and feed them throughout the growing period. Chicks were therefore recaptured and measured every 3–4 days from hatching (age 0 days) until fledging. We measured body mass to the nearest 1 g using a spring balance and foot length (tarsus + middle toe) to the nearest mm using an aluminium wing ruler. Fledging date/age was defined as the first day/age when chicks were able of independent fly for at least 100 m (estimated visually)**.**

The hatching date of 14 out of the 50 juveniles with known migratory status was unknown as we failed to find their nest during incubation. To estimate their hatching date, we back-calculated this parameter from age at ringing using the logistic growth curve built from 273 monitored chicks of known age (Supplementary Figure S1).

### Data analysis

To analyse whether hatching date influenced migratory behaviour, we performed a generalised linear model with a binomial error distribution and logit link function, where juvenile behaviour (migrant or resident) was modelled as a function of Julian hatching date and year. We also tested whether timing of hatching within the season influence their migratory behaviour. For this, each observation (*i*) per year (*j*) was centred around the respective sampled population mean (*Χ*_*ij*_ –*‾Χ*_*j*_) (hereafter, relative hatching date). Then, we used the same model structure with juvenile behaviour as response variable and relative hatching date as predictor. For these analyses, 50 juveniles (31 migrants and 19 residents) with known hatching date were considered.

We explored growth rate for foot length and body mass and assessed whether chick growth was best described by logistic growth, *y*_t_ = *y*_∞_/(1 + exp(− *k*(*t* − *T*_i_))), or Gompertz growth curve, *y*_t_ = *y*_∞_ x exp(− exp(− *k* x (*t* − *T*_i_))), where *y*_t_ is the biometric response, *t* is age, *y*_∞_ is the asymptotic value of response variable, *k* is the growth rate constant and *T*_i_ is the age at the inflection point. For logistic growth, the inflection point occurs at *y*(*T*_i_) = *y*_∞_/2, and for Gompertz at *y*(*T*_i_) = *y*_∞_ /e. Then, we investigated differences on each growth parameter between migrant (n = 14) and resident (n = 14) juveniles considering only those that were measured at least twice and only including measurements up to 35 days of age (mean fledging age 34.4 days ± 3.9 SD, n = 14). We used nonlinear mixed models, with chicks as a random effect to account for pseudo-replication. For the random structure, we only allowed chicks to vary randomly with respect to their asymptotic size (*y*_∞_), as allowing other parameters to vary resulted in lack of model convergence. We then compared models with and without migratory behaviour effect on *y*_∞_, *k* and *T*_i_, and selected the most parsimonious model, which is the model with the fewest parameters within 2 ΔAIC_c_ of the top model^36^.

In order to investigate the potential effects of fledging date on migratory behaviour, we built a logistic regression model with Julian fledging date as the predictor and juvenile migratory behaviour as response variable. We added year as fixed effect to control for potential annual variation, but year was not significant and was therefore excluded. For this analysis, 14 juveniles (8 residents and 6 migrants) with known fledging date were considered.

To explore the influence of parental strategy on juvenile migratory behaviour, we constructed two generalised linear models with a binomial error distribution and logit link function, where juvenile migratory behaviour was modelled as a function of maternal (n = 21) or paternal strategy (n = 22). Sample size differs between models as for a few pairs the behaviour of only one of the adults was known. In addition, we performed a t-test to examine whether breeding phenology (mean relative and absolute hatch date) differed between migrant and resident fathers. All analysis and calculations were performed in R 3.6.3^37^.

### Ethics approval

All animal handling and protocols were carried out in accordance with relevant guidelines and regulations. Ethical approval was provided by Animal Welfare and Ethical Review Board from University of East Anglia. Birds were captured, marked and sampled with permission from the Icelandic Institute of Natural History (Licence No. 3240) and International Wader Study Group (Permit No. 3240).

## Supplementary Information

Supplementary Information.

## Data Availability

Data from the study has been archived at Dryad: 10.5061/dryad.000000010; https://datadryad.org/stash/share/D4AdIdH3KdUcfnq-HgC3EVUHVUm-5ztC1q50Y1m3bYA.
