# Supplementary material for: Paternal effects in the initiation of migratory behaviour in birds
Source: Sci Rep. 2021 Feb 2;11:2782. doi: 10.1038/s41598-021-81274-9 (PMC7854704; doi:10.1038/s41598-021-81274-9)
Supplement: Supplementary file 1 — Supplementary Information. [file 41598_2021_81274_MOESM1_ESM.docx]

**Paternal effects in the initiation of migratory behaviour in birds**

**Méndez V.**^1,*^, **Gill, J.A.**^2^, **Þórisson, B.**^1^, **Vignisson, S.R.**^1, 3^, **Gunnarsson, T.G.**^1^, **Alves J.A.**^1, 4^

^1^ South Iceland Research Centre, University of Iceland, Laugarvatn IS-840, Iceland

^2^ School of Biological Sciences, University of East Anglia, Norwich NR4 7TJ, United Kingdom

^3^ Sudurnes Science and Learning Center, Sandgerði IS-245, Iceland

^4^ Department of Biology & CESAM – Centre for Environmental and Marine Studies, University of Aveiro, Aveiro 3910-193, Portugal

* [veronica@hi.is](mailto:veronica@hi.is)

**Supplementary material**

**Table S1.** Frequency of migrant and resident juveniles in relation to the behaviour of their parents. Only juveniles for which the migratory behaviour of both parents are known are included.

|  |  | **Juvenile migratory behaviour** | |
| --- | --- | --- | --- |
| **Mother** | **Father** | **Migrant** | **Resident** |
| Migrant | Migrant | 5 | 0 |
| Migrant | Resident | 0 | 6 |
| Resident | Migrant | 1 | 0 |
| Resident | Resident | 1 | 2 |

**Table S2.** Total of individuals with known migratory behaviour grouped by age and method used to determine it.

| **Method** | **Behaviour** | **Adults** | **Chicks** |
| --- | --- | --- | --- |
| Observation | Migrant | 121 | 31 |
|  | Resident | 106 | 19 |
| Stable isotopes | Migrant | 232 | - |
|  | Resident | 121 | - |

**Table S3.** Model selection results of logistic and Gompertz growth curves for foot length (tarsus + toe) and body mass testing for differences between migrant and resident juveniles on each growth parameter (*y*_∞_, *k* and *T*_i_). The most parsimonious model is shown in bold.

|  | Model | Effect of juvenile migratory behaviour | df | logLik | AICc | ΔAICc | Akaike weight |
| --- | --- | --- | --- | --- | --- | --- | --- |
| Foot length (mm) | Logistic | **-** | **6** | **-271.19** | **555.24** | **0.00** | **0.20** |
|  |  | *y*_∞_ | 7 | -270.10 | 555.37 | 0.13 | 0.19 |
|  |  | *T*_i_ | 7 | -270.58 | 556.33 | 1.09 | 0.11 |
|  |  | *k* | 7 | -270.83 | 556.82 | 1.58 | 0.09 |
|  |  | *y*_∞_, *T*_i_ | 8 | -269.93 | 557.37 | 2.14 | 0.07 |
|  |  | *y*_∞_, *k* | 8 | -270.10 | 557.71 | 2.47 | 0.06 |
|  |  | k, *T*_i_ | 8 | -270.31 | 558.13 | 2.89 | 0.05 |
|  |  | *y*_∞_, *k*, *T*_i_ | 9 | -269.93 | 559.77 | 4.53 | 0.02 |
| Mass (g) | Gompertz | **-** | **6** | **-466.23** | **945.33** | **0.00** | **0.38** |
|  |  | *y*_∞_ | 7 | -465.83 | 946.83 | 1.49 | 0.18 |
|  |  | *T*_i_ | 7 | -466.10 | 947.36 | 2.03 | 0.14 |
|  |  | *k* | 7 | -466.23 | 947.63 | 2.30 | 0.12 |
|  |  | *y*_∞_, *T*_i_ | 8 | -465.81 | 949.13 | 3.80 | 0.06 |
|  |  | *y*_∞_, *k* | 8 | -465.82 | 949.16 | 3.83 | 0.06 |
|  |  | k, *T*_i_ | 8 | -465.90 | 949.32 | 3.99 | 0.05 |
|  |  | *y*_∞_, *k*, *T*_i_ | 9 | -465.80 | 951.52 | 6.18 | 0.02 |

**Table S4. Average fledging age per year and juvenile migratory behaviour**.

| **Behaviour** | **Year** | **N** | **Range** | **Mean age** | **SE** |
| --- | --- | --- | --- | --- | --- |
| Resident | 2015 | 1 | 30 | 30.00 | NA |
| Migrant | 2016 | 3 | 31-33 | 32.33 | 0.67 |
| Resident | 2016 | 5 | 34-44 | 38.00 | 1.90 |
| Migrant | 2017 | 3 | 30-35 | 32.67 | 1.45 |
| Resident | 2017 | 2 | 33-34 | 33.50 | 0.50 |

**Table S5. Results from the generalised linear model testing for the effect of hatching date on the likelihood of juveniles becoming migrant or resident. Only chicks with known hatching date are included (19 migrants and 17 resident).**

| **Predictor** | **Estimate** | **Std. Error** | **z value** | ***p*** |
| --- | --- | --- | --- | --- |
| (Intercept) | 1.27 | 7.12 | 0.18 | 0.86 |
| Absolute hatching date | -0.01 | 0.04 | -0.22 | 0.83 |
| Year 2016 | 0.68 | 1.07 | 0.64 | 0.52 |
| Year 2017 | -0.21 | 1.09 | -0.19 | 0.85 |
| Year 2018 | -17.29 | 2282.11 | -0.01 | 0.99 |

**Figure S1.** Logistic growth curve built from 273 monitored chicks of known age which was used to estimate hatching date of those chicks which were only caught and ringed at a later age; *y*_t_ = 97.14/(1 + exp(-0.10(*t* – (-0.78)))), where *y*_t_ is the biometric response and *t* is the age.

**
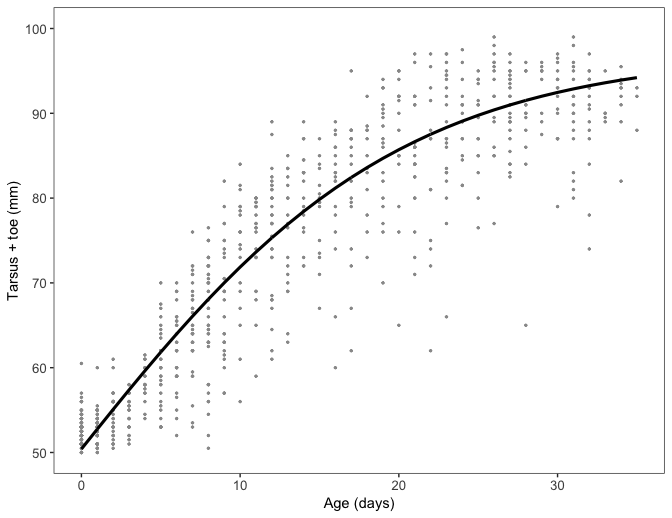
**
